# Deleterious variants in *CRLS1* lead to cardiolipin deficiency and cause an autosomal recessive multi-system mitochondrial disease

**DOI:** 10.1093/hmg/ddac040

**Published:** 2022-02-11

**Authors:** Richard G Lee, Shanti Balasubramaniam, Maike Stentenbach, Tom Kralj, Tim McCubbin, Benjamin Padman, Janine Smith, Lisa G Riley, Archana Priyadarshi, Liuyu Peng, Madison R Nuske, Richard Webster, Ken Peacock, Philip Roberts, Zornitza Stark, Gabrielle Lemire, Yoko A Ito, Care4Rare Canada Consortium, Kym M Boycott, Michael T Geraghty, Jan Bert van Klinken, Sacha Ferdinandusse, Ying Zhou, Rebecca Walsh, Esteban Marcellin, David R Thorburn, Tony Rosciolli, Janice Fletcher, Oliver Rackham, Frédéric M Vaz, Gavin E Reid, Aleksandra Filipovska

**Affiliations:** Telethon Kids Institute, Northern Entrance, Perth Children's Hospital, Nedlands, WA 6009, Australia; Harry Perkins Institute of Medical Research, QEII Medical Centre, Nedlands, WA 6009, Australia; ARC Centre of Excellence in Synthetic Biology, Centre for Medical Research, The University of Western Australia, QEII Medical Centre, Nedlands, WA 6009, Australia; Genetic Metabolic Disorders Service, The Children’s Hospital at Westmead, Sydney, NSW 2145, Australia; Discipline of Genomic Medicine, Sydney Medical School, University of Sydney, Sydney, NSW 2006, Australia; Telethon Kids Institute, Northern Entrance, Perth Children's Hospital, Nedlands, WA 6009, Australia; Harry Perkins Institute of Medical Research, QEII Medical Centre, Nedlands, WA 6009, Australia; ARC Centre of Excellence in Synthetic Biology, Centre for Medical Research, The University of Western Australia, QEII Medical Centre, Nedlands, WA 6009, Australia; School of Chemistry, The University of Melbourne, Parkville, VIC 3010, Australia; Australian Institute for Bioengineering and Nanotechnology, and Queensland Node of Metabolomics Australia,The University of Queensland, St Lucia, QLD 4072, Australia; Centre for Microscopy, Characterisation and Analysis, The University of WA, Perth, WA 6009, Australia; Discipline of Genomic Medicine, Sydney Medical School, University of Sydney, Sydney, NSW 2006, Australia; Department of Clinical Genetics, The Children’s Hospital at Westmead, Sydney, NSW 2145, Australia; Rare Diseases Functional Genomics, Kids Research, The Children’s Hospital at Westmead and Children’s Medical Research Institute, Sydney, NSW 2145, Australia; Discipline of Child and Adolescent Health, University of Sydney, Sydney, NSW 2145, Australia; Discipline of Child and Adolescent Health, University of Sydney, Sydney, NSW 2145, Australia; Neonatal Intensive Care Unit, Westmead Hospital, Sydney, NSW 2145, Australia; School of Chemistry, The University of Melbourne, Parkville, VIC 3010, Australia; School of Chemistry, The University of Melbourne, Parkville, VIC 3010, Australia; Department of Paediatrics, University of Melbourne, VIC 3052, Australia; Kids Neuroscience Centre, The Children’s Hospital at Westmead, Sydney, NSW 2145, Australia; General Paediatric Medicine, The Children's Hospital at Westmead, Sydney, NSW 2145, Australia; Heart Centre for Children, The Children's Hospital at Westmead, Sydney, NSW 2145, Australia; University of Melbourne, Parkville, VIC 3052, Australia; Australian Genomics, Melbourne, VIC 3052, Australia; Victorian Clinical Genetics Services, Melbourne, VIC 3052, Australia; Children’s Hospital of Eastern Ontario Research Institute, University of Ottawa, Ottawa, ON K1H 8L1, Canada; Children’s Hospital of Eastern Ontario Research Institute, University of Ottawa, Ottawa, ON K1H 8L1, Canada; Children’s Hospital of Eastern Ontario Research Institute, University of Ottawa, Ottawa, ON K1H 8L1, Canada; Metabolics and Newborn Screening, Pediatrics, Children’s Hospital of Eastern Ontario, University of Ottawa, Ottawa, ON K1H 8L1, Canada; Department of Clinical Chemistry, Laboratory Genetic Metabolic Diseases, University of Amsterdam, Amsterdam Gastroenterology Endocrinology Metabolism, 1105 AZ Amsterdam, The Netherlands; Core Facility Metabolomics, Amsterdam UMC, University of Amsterdam, 1105 AZ Amsterdam, The Netherlands; Department of Human Genetics, Leiden University Medical Center, 2333ZA Leiden, The Netherlands; Department of Clinical Chemistry, Laboratory Genetic Metabolic Diseases, University of Amsterdam, Amsterdam Gastroenterology Endocrinology Metabolism, 1105 AZ Amsterdam, The Netherlands; NSW Health Pathology, Randwick, NSW 2145, Australia; NSW Health Pathology, Randwick, NSW 2145, Australia; Australian Institute for Bioengineering and Nanotechnology, and Queensland Node of Metabolomics Australia,The University of Queensland, St Lucia, QLD 4072, Australia; University of Melbourne, Parkville, VIC 3052, Australia; Victorian Clinical Genetics Services, Melbourne, VIC 3052, Australia; Murdoch Children’s Research Institute, Melbourne, VIC 3052, Australia; NSW Health Pathology, Randwick, NSW 2145, Australia; Neuroscience Research Australia (NeuRA), University of New South Wales, Sydney, NSW 2145, Australia; NSW Health Pathology, Randwick, NSW 2145, Australia; Telethon Kids Institute, Northern Entrance, Perth Children's Hospital, Nedlands, WA 6009, Australia; Harry Perkins Institute of Medical Research, QEII Medical Centre, Nedlands, WA 6009, Australia; ARC Centre of Excellence in Synthetic Biology, Centre for Medical Research, The University of Western Australia, QEII Medical Centre, Nedlands, WA 6009, Australia; Curtin Medical School, Curtin University, Bentley, WA 6102, Australia; Curtin Health Innovation Research Institute, Curtin University, Bentley, WA 6102, Australia; Department of Clinical Chemistry, Laboratory Genetic Metabolic Diseases, University of Amsterdam, Amsterdam Gastroenterology Endocrinology Metabolism, 1105 AZ Amsterdam, The Netherlands; Core Facility Metabolomics, Amsterdam UMC, University of Amsterdam, 1105 AZ Amsterdam, The Netherlands; Department of Pediatrics, Emma Children's Hospital, Amsterdam UMC, University of Amsterdam, 1105 AZ Amsterdam, The Netherlands; School of Chemistry, The University of Melbourne, Parkville, VIC 3010, Australia; Department of Biochemistry and Pharmacology, The University of Melbourne, Parkville, VIC 3010, Australia; Bio21 Molecular Science and Biotechnology Institute, The University of Melbourne, Parkville, VIC 3010, Australia; Telethon Kids Institute, Northern Entrance, Perth Children's Hospital, Nedlands, WA 6009, Australia; Harry Perkins Institute of Medical Research, QEII Medical Centre, Nedlands, WA 6009, Australia; ARC Centre of Excellence in Synthetic Biology, Centre for Medical Research, The University of Western Australia, QEII Medical Centre, Nedlands, WA 6009, Australia

## Abstract

Mitochondrial diseases are a group of inherited diseases with highly varied and complex clinical presentations. Here, we report four individuals, including two siblings, affected by a progressive mitochondrial encephalopathy with biallelic variants in the cardiolipin biosynthesis gene *CRLS1*. Three affected individuals had a similar infantile presentation comprising progressive encephalopathy, bull’s eye maculopathy, auditory neuropathy, diabetes insipidus, autonomic instability, cardiac defects and early death. The fourth affected individual presented with chronic encephalopathy with neurodevelopmental regression, congenital nystagmus with decreased vision, sensorineural hearing loss, failure to thrive and acquired microcephaly. Using patient-derived fibroblasts, we characterized cardiolipin synthase 1 (CRLS1) dysfunction that impaired mitochondrial morphology and biogenesis, providing functional evidence that the *CRLS1* variants cause mitochondrial disease. Lipid profiling in fibroblasts from two patients further confirmed the functional defect demonstrating reduced cardiolipin levels, altered acyl-chain composition and significantly increased levels of phosphatidylglycerol, the substrate of CRLS1. Proteomic profiling of patient cells and mouse *Crls1* knockout cell lines identified both endoplasmic reticular and mitochondrial stress responses, and key features that distinguish between varying degrees of cardiolipin insufficiency. These findings support that deleterious variants in *CRLS1* cause an autosomal recessive mitochondrial disease, presenting as a severe encephalopathy with multi-systemic involvement. Furthermore, we identify key signatures in cardiolipin and proteome profiles across various degrees of cardiolipin loss, facilitating the use of omics technologies to guide future diagnosis of mitochondrial diseases.

## Introduction

Mitochondria are double membrane organelles that have key roles in energy production, regulation of programmed cell death, lipid metabolism, redox and ion homeostasis and hormone biosynthesis (1,2). The unique lipid and protein composition of the mitochondrial membranes enables the selective transport of ions and metabolites, regulates mitochondrial dynamics by fusion and fission and maintains the membrane potential required for oxidative phosphorylation (OXPHOS). Due to the essential role of OXPHOS in energy generation, mitochondrial membranes have become a key area of interest in mitochondrial disease (MD) and biology. Pathogenic variants in key mitochondrial membrane and lipid regulators have been implicated in various MDs, including *OPA1* (3,4). Furthermore, variants of membrane morphology regulators have also been implicated in pathogenesis of other disease states, such as *CHCHD2* variants in Parkinson’s disease (5,6), and *CHCHD10* variants in frontotemporal dementia and amyotrophic lateral sclerosis (FTD-ALS) (7).

The mitochondrial membrane-specific phospholipid cardiolipin (CL) has emerged as a central player in mitochondrial pathologies. CL is a key component of the mitochondrial inner membrane that facilitates the assembly and stability of large protein complexes central to mitochondrial function, such as the OXPHOS system and the mitochondrial contact site and cristae organizing system (MICOS) (8). Mutations in the CL remodelling enzyme Tafazzin (encoded by *TAFAZZIN*) cause the multi-system disease Barth syndrome (BTHS), which is characterized by cardiomyopathy, skeletal myopathy, altered growth, 3-methylglutaconic aciduria and neutropenia (9). Furthermore, changes in CL abundance and acyl-chain composition have been identified in many diseases including type 2 diabetes mellitus, Parkinson’s disease, heart disease and in ageing (10,11), indicating that altered CL levels and composition are risk factors for human disease. The cardiolipin synthase 1 (CRLS1) enzyme has a major role in CL biosynthesis by converting phosphatidylglycerol (PG) into CL (12,13). Deletion of mouse *Crls1* in cells (14) results in loss of CL and models mitochondrial defects in disease (15), such as impaired coordination of mitochondrial protein synthesis (14) and OXPHOS biogenesis (16). However, pathogenic variants in the nuclear-encoded *CRLS1* gene have not been identified in patients to date and the exact mechanisms linking CL to a wide range of pathologies are not clear.

Here, we have identified a homozygous *CRLS1* p.(Ile109Asn) variant in three individuals (Family 1 Patient II:4, also referred to as Subject 1; Family 2 Patients II:1 and II:3) presenting with a new form of MD characterized by an evolving pattern of cardiomyopathy, encephalopathy, bilateral auditory neuropathy spectrum disorder, bull’s eye maculopathy, diabetes insipidus, autonomic instability and low complex IV activity in skeletal muscle. In addition, we identified segregating heterozygous *CRLS1* p.(Ala172Asp) and p.(Leu217Phe) variants in a fourth individual (Family 3 Patient II:1, referred to as Subject 2) presenting with developmental regression beginning in late infancy, with acquired microcephaly, sensorineural hearing loss and impaired vision. Using patient fibroblasts, we validated the pathogenicity of the *CRLS1* variants through combined lipidomic, proteomic and cell microscopy approaches and used both patient fibroblasts and *Crls1* knockout cell lines (14), to show that synergistic stress responses driven by both the mitochondria and endoplasmic reticulum (ER) vary based on the degree of CL loss. Through these combined approaches, we have established *CRLS1* as a new disease gene driving MD pathogenesis and identified diagnostic applications of omics technologies for CL-related disorders.

**Figure 1:**
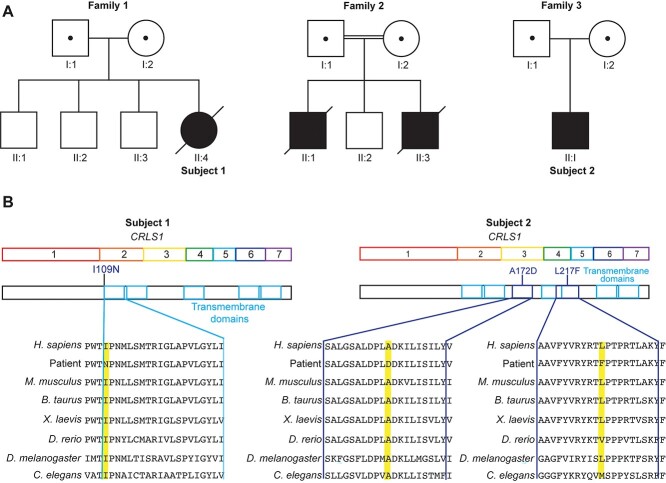
Identification of novel *CRLS1* variants in multisystem MD patients. (**A**) Pedigrees of three families showing consanguinity in Family 2. The affected individuals are represented with a black symbol, and their parents are carriers for the identified variants (symbols containing a dot represent a heterozygous carrier status). (**B**) Schematic representation of the *CRLS1* gene and its encoded protein, with transmembrane domains annotated showing the variant locations. Multiple sequence alignment for CRLS1 in the patients and in diverse species where the variant amino acid location is highlighted in yellow.

## Results

### Clinical characteristics of the patients

The patient from Family 1 (II:4, Subject 1) (Fig. 1A) was born to non-consanguineous Lebanese parents at 34 weeks, was small for gestational age (2.15 kg) and had non-immune hydrops fetalis and bilateral pleural effusions necessitating respiratory support after birth. Left-ventricular non-compaction with severe biventricular systolic dysfunction was identified on day 2 by echocardiography and evolved to hypertrophic cardiomyopathy by 7 weeks of life. She displayed central hypotonia and myoclonic jerks within the first few days of life and subsequently became encephalopathic with bulbar dysfunction following a catastrophic cardiac arrest at 8 weeks. She developed bull’s eye maculopathy, bilateral auditory neuropathy, nephrogenic diabetes insipidus and autonomic instability over the next few weeks. Progressive neurological deterioration resulted in redirection of care towards palliation and the patient died at 3.5 months. Magnetic resonance imaging (MRI) of the brain at 6 weeks and magnetic resonance spectroscopy (MRS) at 10 weeks were both normal (Supplementary Material, Fig. S1). Respiratory chain enzyme (RCE) activities measured in liver and skeletal muscle homogenates identified marked complex IV deficiency in skeletal muscle, with more modest reductions in complexes I and III activities. In conjunction with clinical features, these supported a diagnosis of MD according to the Bernier diagnostic criteria (17) (Table 1 and Supplementary Material, Fig. S2A–D). Conversely, liver homogenates displayed no major changes in RCE activity of any complexes and some minor structural changes (Supplementary Material, Fig. S2E). Additionally, both tissues showed the same level of complex II activity, which is the only complex that does not have a subunit encoded by the mitochondrial DNA (mtDNA) (18).

**Table 1:** Respiratory chain enzyme activity measured in Subject 1 skeletal muscle and liver homogenates

	Complex I		Complex II		Complex II + III		Complex III		Complex IV		Citrate synthase (CS)
	Activity	% of CS activity	Activity	% of CS activity	Activity	% of CS activity	Activity	% of CS activity	Activity	% of CS activity	Activity
Sk muscle (*n* = 9 controls)	10 (19–72)	31	28 (26–63)	80	18 (30–76)	51	9.2 (13–51)	39	**0.75 (3.3–9.1)**	**15**	99 (85–179)
Liver (*n* = 6 controls)	11 (7.8–11.2)	71	79 (54–73)	80	n.t	n.t	11.1 (5.2–10.3)	90	1.35 (0.45–0.87)	123	44 (26–31)

Enzyme activities are displayed along with the control ranges (shown in brackets) and as % activity relative to citrate synthase activity. Values in bold are below the Major Criterion threshold for mitochondrial disease pathology in the Bernier Diagnostic Scheme (17).

The two siblings from Family 2 (II:1 and II:3) were the first and third children born to consanguineous parents (Fig. 1A). Patient II:1 was the result of a pregnancy with decreased amniotic fluid late in the gestation and was delivered at 38 weeks (3.22 kg), requiring resuscitation. He presented with feeding difficulties at day 10 and was admitted to the neonatal intensive care unit (NICU) shortly after for recurrent apnea. He was hypotonic, had bilateral auditory neuropathy and vocal cord palsy. He subsequently followed a progressive neurodegenerative course characterized sequentially by epileptic encephalopathy with bulbar dysfunction, non-epileptic myoclonus, central hypoventilation and dystonia, auditory neuropathy, bull’s eye maculopathy, central diabetes insipidus and autonomic instability with recurrent unexplained fevers. Serial brain MRIs showed progressive cerebral atrophy (Supplementary Material, Fig. S3A–C) with preservation of the brainstem and cerebellum. He required ongoing invasive respiratory support and died at the age of 10 months. Patient II:3 from Family 2 was delivered at 36 weeks of gestation with a birth weight of 2.49 kg and was admitted to the NICU on day two for a choking episode. Echocardiography showed severe biventricular dysfunction requiring inotropic support, which subsequently improved. He developed RSV bronchiolitis at 9 weeks, requiring ventilation, and thereafter became progressively encephalopathic with seizures, bulbar dysfunction and dystonia. In addition, he developed bull’s eye maculopathy, auditory neuropathy, autonomic instability with recurrent unexplained fever and diabetes insipidus. Brain MRI showed subtle loss of brain volume at 3 months (Supplementary Material, Fig. S3D and E). He died at the age of 4 months.

The patient from Family 3 (II:1, Subject 2) (Fig. 1A) was of Caucasian background and evaluated at 3 months for unexplained hypotonia, congenital nystagmus and sensorineural hearing loss. His EEG was normal; however, somatosensory evoked potential (SSEP), visual evoked response (VER), and brainstem auditory evoked response (BAER) were all abnormal. His development was globally delayed and he displayed slowly progressive encephalopathy with further developmental regression in late infancy, acquired microcephaly, sensorineural hearing loss and decreased vision. At ~2.5 years, brain MRI showed cerebral atrophy and developmental assessment showed the patient’s best skills were rolling over, grasping and mouthing objects with the right hand, and making some immature vocalizations. At 19 years, he has a permanent disability with poor functional status and total dependence for all aspects of care.

### Novel variants in *CRLS1* cause mitochondrial dysfunction

Clinical presentation in the patients and RCE activity changes indicated that a mitochondrial defect may be the underlying cause of the disease pathology. mtDNA sequencing in patients from Family 1, II:4 (Subject 1) and Family 2, II:3 identified no causative variants in mtDNA-encoded genes. To investigate pathogenic variants, trio whole exome sequencing (WES) was conducted on the affected individuals and both parents in each family. We identified a novel homozygous missense variant not previously identified in *CRLS1:* Chr20(GRCh38):g.6009794T>A, NM_019095*.5*:c.326T>A mutation in Family 1 and 2, resulting in a p.(Ile109Asn) substitution (Fig. 1B and Supplementary Material, Fig. S4A) The variant was confirmed by Sanger sequencing (Supplementary Material, Fig. S4A and B), and all parents were heterozygous for the *CRLS1* variant; although they are of the same ethnic background, they are not known to be related. *In silico* prediction programs, including CADD, MutationTaster, SIFT and Polyphen-2, all predict this missense change to have a deleterious effect on the CRLS1 protein. The c.326T>A variant is rare in gnomAD v.2.1.1, with only one allele identified (1/251 152 alleles) and an allele frequency of 0.00039% (19). The CRLS1 Ile109Asn substituted amino acid is highly conserved across a wide range of animal species (GERP score of 5.4) (Fig. 1B and Supplementary Material, Fig. S4C) and the substitution of a non-polar isoleucine with a polar asparagine at the start of a highly conserved transmembrane domain could affect stability or membrane localization of CRLS1 (Fig. 1B). WES analysis in the proband from Family 1 also identified a variant in *LYRM4:* NM_001164840.2: c.82T>C resulting in a p.(Tyr28His) substitution (Supplementary Material, Fig. S4B), which was not found in the affected individuals from Family 2. The c.82T>C variant is absent from gnomAD (19) and is highly conserved across a wide range of animal species (GERP score of 5) (Fig. 1B and Supplementary Material, Fig. S4C). The *LYRM4* variant results in a tyrosine to histidine substitution; however, this variant is situated upstream of the predicted cleavage site of the mitochondrial targeting sequence (MTS) (amino acid 32, predicted by PSORT) so would not likely be present in the mature form of the protein (Supplementary Material, Fig. S4C). Furthermore, this altered residue was not predicted to change the PSORT-determined MTS cleavage site and does not cause an unfavourable polarity change that would impede mitochondrial localization. Functional studies did not support a role for this protein in the patient’s disease (Supplementary Material, Fig. S4D–G) (20,21); therefore, we conclude the *LYRM4* variant is not contributing to the clinical presentation in Family 1.

Trio exome sequencing identified segregating heterozygous variants in *CRLS1* in the proband from Family 3 (Subject 2): NM_019095.6: c.515C>A p.(Ala172Asp) and c.649C>T p.(Leu217Phe) (Fig. 1B). The maternally inherited c.515C>A variant occurs at a conserved site (GERP score of 6) and is absent from gnomAD (19). *In silico* prediction programs, including CADD, MutationTaster, SIFT and Polyphen-2, all predict this missense change to have a deleterious effect on the CRLS1 protein. The c.649C>T variant was paternally inherited, occurs at a conserved site (GERP score of 6) and is rare in gnomAD v.2.1.1, with only four alleles identified (4/245 604 alleles) and an allele frequency of 0.0016% (19). *In silico* prediction programs, including CADD, MutationTaster, SIFT and Polyphen-2, all predict this missense change to have a deleterious effect on the CRLS1 protein. Neither variant has been observed in the homozygous state in the gnomAD database. No other variants in known or novel genes have been retained as plausible candidates by exome analysis. Amino acids 172 and 217 are highly conserved across animal species (Fig. 1B). Both substitutions introduce bulkier sidechains, the aspartate substitution introduces a charge and the phenylalanine substitution is more hydrophobic but does not alter amino acid polarity, suggesting that the p.(Leu217Phe) variant would likely not affect the CRLS1 protein as severely as the p.(Ile109Asn) variant. This is consistent with the clinical presentation of each variant, whereby the p.(Ile109Asn) variant caused early lethality, while the p.(Ala172Asp) and p.(Leu217Phe) variants were compatible with life, albeit associated with severe developmental defects.

**Figure 2:**
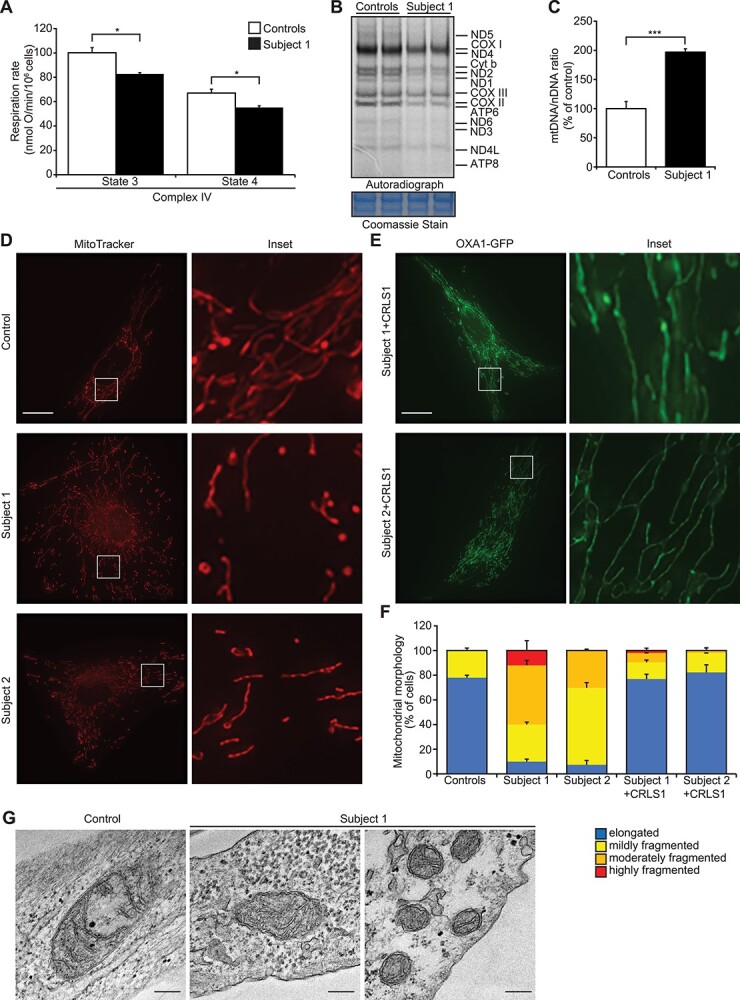
*CRLS1* p.(Ile109Asn) variant causes mitochondrial membrane ultrastructural defect and reduction in OXPHOS. (**A**) Oxygen consumption of specific complexes under phosphorylating (state 3) and non-phosphorylating (state 4) conditions was measured in controls from two paediatric individuals and Subject 1 fibroblasts using an OROBOROS high-resolution respirometer. Data are from at least three independent biological experiments. (**B**) De novo mitochondrial protein synthesis was measured by ^35^S methionine/cystine incorporation. 17.5 μg of cell lysates from control and Subject 1 fibroblasts were separated by SDS-PAGE and visualized by autoradiography. Coomassie stained gels are shown as loading controls. (**C**) MtDNA copy number was measured using quantitative real-time PCR in control and Subject 1 fibroblasts. Data are presented as mean ± SEM. *P* < 0.05, ^*^; *P* < 0.001, ^*^^*^^*^. (**D**) Mitochondrial network morphology was visualized in two control and Subjects 1 and 2 fibroblasts using MitoTracker Orange and imaged using confocal fluorescence microscopy. Scale bar is 10 μm. (**E**) Mitochondrial morphology of Subjects 1 and 2 cells expressing wild-type CRLS1 visualized by examining co-expressed OXA1-GFP fluorescence. (**F**) Qualitative scoring of mitochondrial morphology (*n* > 50) for all cells. Details of morphology classifications are described in (14). (**G**) Transmission electron micrographs of control and Subject 1 fibroblasts mitochondria. Scale bar is 200 nm.

To investigate how the *CRLS1* p.(Ile109Asn) variant contributes to disease pathogenesis, mitochondrial respiration and OXPHOS protein stability were measured in fibroblasts from Subject 1 and a healthy control (Fig. 2A and Supplementary Material, Fig. S4E–G), as these were impacted in mouse *Crls1* knockout cells (14). High-resolution respirometry showed a 20% reduction in complex IV activity in the ATP-generating (phosphorylating state 3) and non–ATP-generating (non-phosphorylating state 4) conditions (Fig. 2A), while both states 3 and 4 respiration of complex I were comparable between the control and Subject 1 fibroblasts (Supplementary  Material, Fig. S4E). Immunoblotting of OXPHOS proteins isolated from control and Subject 1 fibroblasts showed increased levels of complex I, III, IV and V subunits, while no change was observed in the complex II subunit (Supplementary Material, Fig. S4F). OXPHOS complex formation of complexes I, III and V showed a minor increase consistent with western blots of the individual subunits; however, complex IV levels were reduced in the patient fibroblasts (Supplementary Material, Fig. S4G). Loss of CL reduces the rate of mitochondrial protein synthesis by decreasing the association of the translating mitoribosome with the inner mitochondrial membrane (14). We showed that Subject 1 fibroblasts had reduced de novo mitochondrial translation rates for all 13 mitochondrially encoded proteins (Fig. 2B), despite an ~2-fold increase in mtDNA copy number (Fig. 2C). An increase in mtDNA levels is a compensatory response to reduced mitochondrial protein synthesis that was found in cardiolipin synthase knockout (*Crls1^KO^*) cells (14), implicating the *CRLS1* p.(Ile109Asn) variant in disease pathogenesis.

CL helps maintain mitochondrial network integrity (14); therefore, we examined mitochondrial morphology in fibroblasts from Subjects 1 and 2 compared to controls. In both subjects, the mitochondrial network was fragmented towards the periphery of the cell but retained some interconnected tubular network structures around the nucleus, with Subject 1 showing more severe fragmentation than Subject 2 (Fig. 2D). Rescue experiments with transient expression of the wild-type CRLS1 in both Subjects 1 and 2 fibroblasts restored the reticular morphology to that of control fibroblasts, with long-tubular structures distributed evenly throughout the cell (Fig. 2E and F), indicating that the *CRLS1* variants were responsible for the impaired mitochondrial morphology. Transmission electron microscopy (TEM) showed that Subject 1 mitochondria were smaller, rounder and contracted compared to control mitochondria (Fig. 2G). The patient mitochondria had highly disordered cristae morphology and large areas devoid of cristae, indicating that cristae folding was impaired (Fig. 2G), similar to defects found in BTHS patient cells (15). The rescue experiments indicated that the *CRLS1* p.(Ile109Asn) variant impaired CL-associated regulation of mitochondrial membranes, likely resulting in the observed OXPHOS defects.

**Figure 3:**
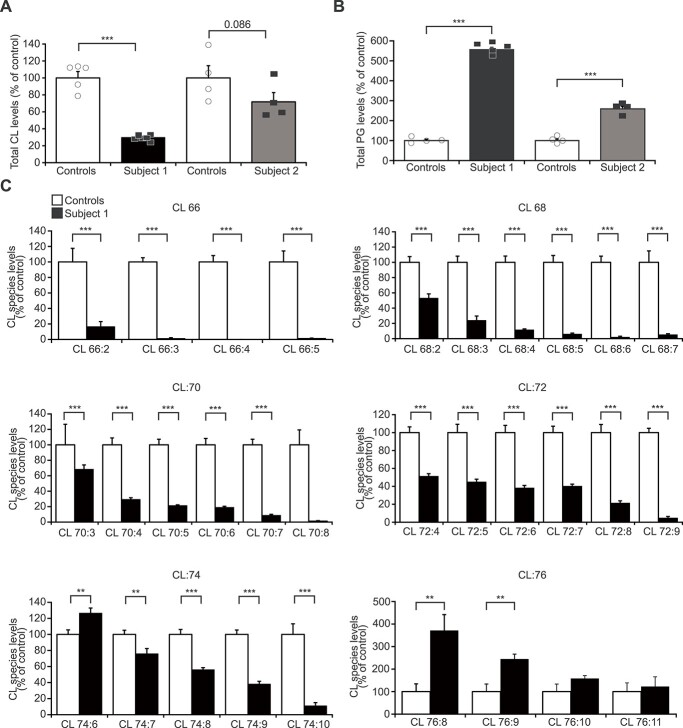
The *CRLS1* variants alter CL abundance and acyl-chain profile. (**A**) Total cardiolipin (CL) levels in control and patient fibroblasts determined by mass spectrometry–based lipidome analysis from at least four independent biological samples. Data are displayed as percent of respective control levels. (**B**) Phosphatidylglycerol (PG) levels in controls and Subjects 1 and 2 fibroblasts. (**C**) Cardiolipin acyl-chain composition of Subject 1 cells. Data are percent change of each species compared to control levels and shown as mean ± SEM. FDR < 0.05, ^*^; FDR < 0.01, ^*^^*^; FDR < 0.001, ^*^^*^^*^.

### *CRLS1* variants reduce CL abundance and alter the CL acyl-chain composition

To validate the effects of the CRLS1 variants on CL, we examined CL abundance and acyl-chain composition profiles of control and patient fibroblasts from Subjects 1 and 2 using mass spectrometry–based lipidome analysis (14,22). We found a significant reduction in total cell CL in Subject 1 and a trend towards reduced CL in Subject 2 (Fig. 3A), consistent with the less damaging effect of the variants in this patient on the function of CRLS1. PG levels were significantly increased in the fibroblasts of both Subjects 1 and 2 (Fig. 3B), with Subject 1 showing a more pronounced increase than Subject 2. The PG/CL ratio was consequently increased by ~15-fold and ~4-fold in Subjects 1 and 2 fibroblasts, respectively, compared to controls, with a similar change shown in Subject 2 lymphocytes (Supplementary Material, Fig. S5A). This demonstrates an accumulation of the substrate of CRLS1 and shows an imbalance in the CL synthesis pathway in both patients that was reflective of the severity of the disease. The CL acyl-chain composition of Subject 1 cells showed more significant reduction in shorter acyl-chain length CL species, and particularly those containing highly unsaturated acyl-chains (Fig. 3C). The CL acyl-chain composition in the fibroblasts from Subject 2 showed significant reductions in the levels of multiple highly unsaturated acyl-chain containing CL species, such as 72:7 and 72:8, accompanied by increases in more saturated species (Supplementary Material, Fig. S5B), changes that are consistent with Subject 1 and defects in CL biosynthesis and remodelling. These data functionally validate the pathogenicity of the *CRLS1* variants, illustrating that they cause decreased CL levels, altered acyl-chain composition and elevated levels of PG.

### Loss of cardiolipin elicits concerted ER and mitochondrial stress responses

**Figure 4:**
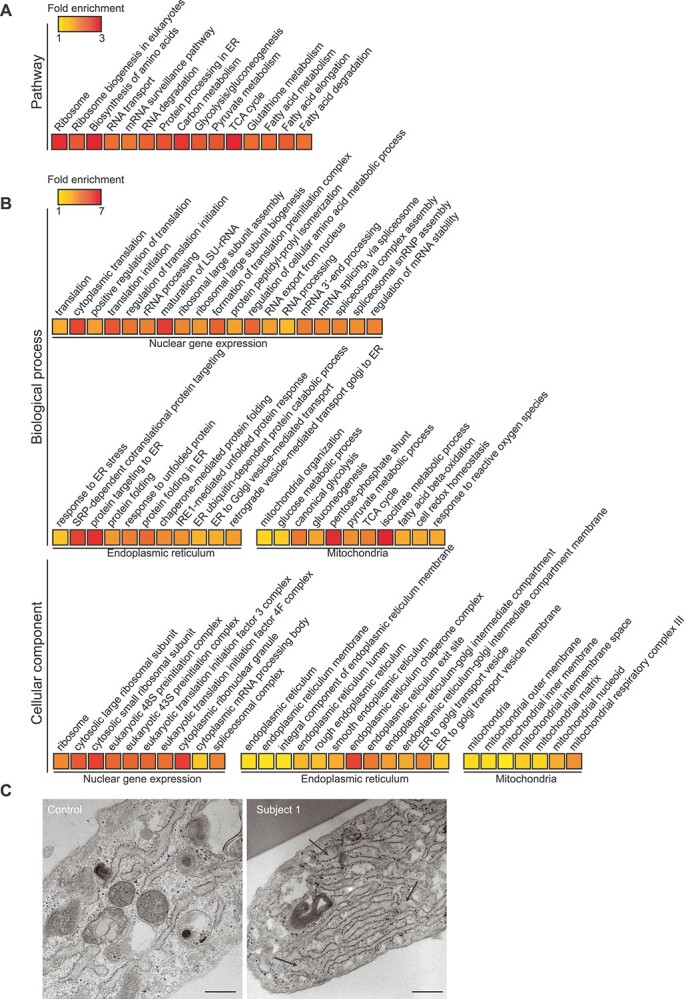
*CRLS1* p.(Ile109Asn) cells display an ER-driven gene expression stress response. Fold enrichment of (**A**) KEGG pathways and (**B**) biological process and cellular compartment gene ontologies determined by quantitative whole-cell proteomics performed on controls and Subject 1 fibroblasts. Significance of pathway enrichment determined at FDR < 0.01. Data are from five independent biological samples. (**C**) Transmission electron micrographs of control and patient fibroblast endoplasmic reticulum. Scale bar is 200 nm and arrows indicate hypertrophic ER morphology.

Severe loss of CL leads to concerted transcriptional stress response mediated by increases in mitochondrial and ER transcription factors (14). Whole-cell quantitative proteomics showed changes in gene expression pathways, protein processing in the ER and carbon metabolism pathways linked to mitochondrial metabolite homeostasis and energy production outside of the OXPHOS complexes in Subject 1 compared to control fibroblasts (Fig. 4A). In Subject 1 cells, we found widespread changes in gene ontologies (GOs) relating to cytoplasmic translation, including changes in small and large cytoplasmic ribosomal subunits, translation initiation, elongation, rRNA processing and mRNA-related processes like mRNA stability, processing and splicing (Fig. 4B). The *CRLS1* p.(Ile109Asn) variant also elicited an ER–stress response, related to co-translational ER protein import, chaperone-mediated protein folding and ER protein transport in the patient cells (Fig. 4B). This large ER response at the protein level led us to examine ER morphology in patient cells using TEM (Fig. 4C). Subject 1 cells displayed an increased abundance of highly hypertrophic ER, which is a morphological response to the induction of ER–stress pathways (23). This indicates that the *CRLS1* p.(Ile109Asn) variant elicits a compensatory response to impaired mitochondrial function through an ER-supported increase in nuclear-gene expression.

**Figure 5:**
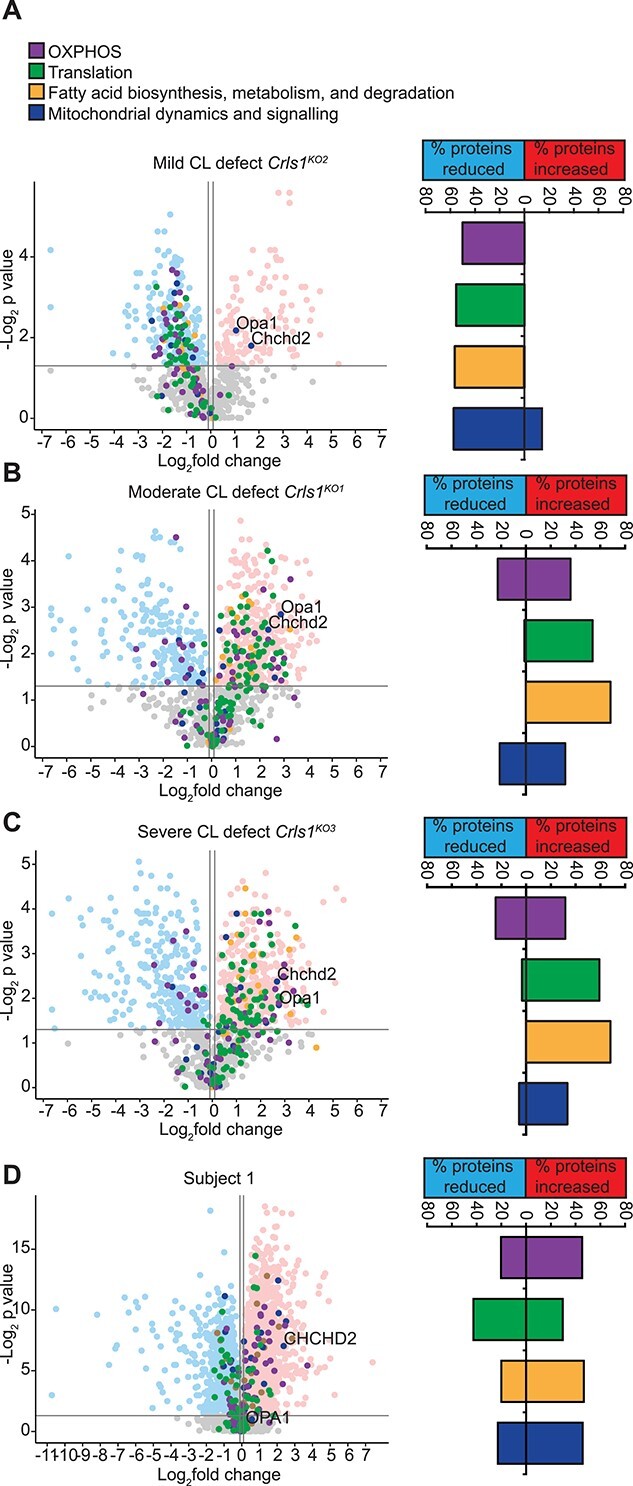
Mitoproteome profiles are altered based on severity of the CL defect. Volcano plots of quantitative mitoproteome analyses showing the percent of detected proteins significantly changing for each highlighted pathway for (**A**) mild (*Crls1^KO2^*), (**B**) moderate (*Crls1^KO1^*), (**C**) severe (*Crls1^KO3^*) and (**D**) Subject 1 cells relative to their respective control cells. Data are from five independent biological samples.

Next, we examined the mitochondrial proteome in Subject 1 cells as well as mouse *Crls1* knockout cell lines (14), to understand the mitochondrial response to varying degrees of CL insufficiency (Fig. 5A–D). The three *Crls1* knockout fibroblast cell lines display minor (*Crls1^KO2^*), moderate (*Crls1^KO1^*) and severe (*Crls1^KO3^*) mitochondrial fragmentation as a consequence of impaired CL biosynthesis related to differentially reduced CL levels (14). Moderate and severe loss of CL caused significant increases in identified proteins predominantly involved in mitochondrial translation, and fatty acid biosynthesis, metabolism and degradation (Fig. 5B and C), followed by OXPHOS and mitochondrial dynamics and signalling proteins (Fig. 5B and C). In contrast, the cells with the mild CL defect showed a robust reduction of all of these mitochondrial processes (Fig. 5A), indicating a divergence in stress responses to varying degrees of CL insufficiency. Interestingly, known regulators of mitochondrial dynamics, OPA1 and CHCHD2, were some of the few proteins that were increased in all three KO lines, indicating a common, mitochondrial membrane–related stress response to CL insufficiency. In the Subject 1 fibroblasts, changes in OXPHOS, fatty acid biosynthesis, metabolism and degradation, and mitochondrial dynamics and signalling proteins were similar to *Crls1* knockout cells that lack CL and have moderate or severe CL defect (Fig. 5D), consistent with the severity of the CL defect in Subject 1. However, the changes in proteins involved in mitochondrial translation were more similar to cells with residual CL levels like the Subject 1 cells (Fig. 5D), which reduced the association of the mitoribosome with the membrane and lowered the rate of translation (14). While OPA1 levels were unchanged in Subject 1 cells, CHCHD2 levels were increased similarly to all three *Crls1* knockout cell lines (Fig. 5D), suggesting that CHCHD2 may be an important marker of CL deficiency and mitochondrial membrane related stress, which can occur independently or in conjunction with the ER-driven gene expression response.

## Discussion

Here, we identified three pathogenic variants in *CRLS1* in three families presenting with multi-system MD with varying degrees of severity from premature death in three infants to permanent disability into young adulthood, highlighting the essential role of CL in mitochondrial function. Our data support that biallelic deleterious variants in the *CRLS1* gene cause an autosomal recessive mitochondrial disease and highlight the essential role of CL in mitochondrial function. Further support for the pathogenic role of the *CRLS1*:c.326T>A variant resulting in a Ile109Asn substitution by functional analyses of patient fibroblasts showed distinct loss of CL and fragmented mitochondrial morphology that is common to cell lines where *Crls1* has been knocked out (14). Abnormal cristae morphology found in the patient cells has been previously observed in *Crls1^KO^* cell lines and BTHS patient lymphoblasts (14,15), and here we show that the defects caused by the *CRLS1* variants were rescued by expression of wild-type CRLS1 in the patient cells. Furthermore, the role of the *CRLS1* p.(Ile109Asn) variant in MD was supported by the reduction in complex IV-stimulated respiration and compensatory increase in OXPHOS protein levels and mtDNA copy number, in response to decreased mitochondrial protein synthesis. The uncoordinated mitochondrial translation rates resulting from an inability of the mitoribosome and OXA1 to interact has been shown to increase OXPHOS subunit expression and mtDNA copy number in an attempt to overcome defects in RCE assembly (14). Furthermore, we show that the segregating heterozygous *CRLS1* p.(Ala172Asp) and p.(Leu217Phe) variants alter CL acyl-chain profile and reduce CL abundance, which results in a debilitating, albeit non-lethal to date, MD.

The reduced levels of total CL confirmed that the p.(Ile109Asn) variant caused impaired CL biosynthesis. The increased levels of PG in both patients suggest that cells from Subject 2 are able to compensate for the less severe defects in CRLS1 function resulting from the p.(Ala172Asp) and p.(Leu217Phe) segregating heterozygous variants by elevating the levels of CL precursors, but this mechanism cannot overcome the more severe defects seen in Subject 1 cells. Despite this compensatory mechanism, both patients displayed a shift in CL acyl-chain composition towards longer acyl-chain length and more saturated CL species. CL defects in both patients may contribute to impaired TAFAZZIN-mediated transacylation activity that incorporates a linoleoyl acyl chain from phosphatidylcholine (PC) to monolysocardiolipin (MLCL) to form mature CL (24). Both yeast Taz1p and mammalian TAFAZZIN have been shown to associate with mitochondrial membranes and exist in higher order assemblies at the inner membrane (25,26), whose assembly is impaired by known TAFAZZIN activity-reducing BTHS point mutations (25). The key role of CL in facilitating both the formation of large protein complexes and proper folding of the mitochondrial inner membrane cristae suggests that severe depletion of CL in p.(Ile109Asn) cells could impair the formation of higher order TAFAZZIN assemblies, as well as the possibility that mutations in or adjacent to CRLS1 transmembrane domains may impede the ability of newly synthesised CL to interact with TAFAZZIN. Reduced TAFAZZIN activity could result in CL modification relying more on other pathways, such as lysocardiolipin acyltransferase 1 (LCLAT1)-mediated remodelling, which has been shown to preferentially incorporate very long polyunsaturated fatty acids, such as arachidonic acid and docosahexaenoic acid, into CL, which would result in the bias towards longer acyl-chain species observed in both patients. Excess incorporation of these species into CL has been previously linked to impaired mitochondrial fusion and OXPHOS function (27,28) and could further potentiate the mitochondrial dysfunction resulting from impaired CRLS1 function.

The pathology presenting in a subset of tissues in the p.(Ala172Asp) and p.(Leu217Phe) segregating heterozygous variants patient compared to the p.(Ile109Asn) variant patient may reflect certain tissues having more specific requirements for CL acyl-chain length and unsaturation to fine-tune their mitochondrial function in addition to CL acyl-chain abundance. This presents another element of CL insufficiency that can contribute to human disease. The clinical presentation and affected tissues of the CRLS1 patients (neurological and cardiac) vary significantly from BTHS patients (cardiac, muscle, immunological) despite the similar molecular and biochemical changes, emphasising how different defects in CL biosynthesis may have drastically varied disease pathogeneses, possibly depending on tissue-specific CL requirements and profiles (29). This is further exemplified in our three cell models that have specific molecular signatures and stress responses to varied CL insufficiency. The clinical features of the p.(Ile109Asn) variant patients and the patient with the p.(Ala172Asp) and p.(Leu217Phe) segregating heterozygous variants illustrate a disease spectrum for CRLS1 dysfunction, emphasising the need to understand the pathogenesis of multi-system CL-related disorders. The differences between the patient proteomes and those of the *Crls1^KO^* cells are a consequence of the type of mutations in CRLS1, and different cell types. The *Crls1^KO^* cells that lack CL, have a more profound effect on overall OXPHOS function causing complete fragmentation of the mitochondrial network (14). The patients’ fibroblasts have varying CL levels and acyl-chain profiles that can maintain greater levels of OXPHOS function than the *Crls1^KO^* cells and show only partial fragmentation of the mitochondrial network. However, the patients’ CL defects appear to be more detrimental to OXPHOS function in tissues such as the heart, skeletal muscle and brain resulting in early-onset death for Subject 1 and debilitating neurological defects in Subject 2, indicating the variable role of CL abundances and acyl-chain profiles in regulating mitochondrial function across cells with different metabolic environments. Nevertheless, the similarity in mitochondrial protein changes between patient and severe CL defect cells indicates that this ER–stress response drives these mitochondrial compensatory changes and also explains the differential mitoproteome response between cells with mild and severe CL defects, as cells with mild CL defect do not have the same transcriptional changes as cells with a severe CL defect (14). Mild mitochondrial dysfunction often manifests in transcriptional activation of stress responses in an effort to rescue or compensate for a defect (30), whereas a severe mitochondrial dysfunction results in proteome changes that often lead to organismal decline (31,32), as it has been observed for significant CL loss in the patient with the p.(Ile109Asn) variant and the *Crls1^KO^* cells (14). Different ER–mitochondria–associated membrane (MAM) enzymes have been found to influence mitochondrial CL composition, including LCLAT1 (27,33). The observed ER stress responses may also contribute to the lipid-related compensatory mechanisms in CL deficiency and drive the shift in CL profile observed in the patients.

## Conclusions

The characterization of the *CRLS1* variants identified in MD pathogenesis advances our understanding of the role of CL in human disease. Combining omics technologies with clinical examination can serve as the next step in MD management, facilitating the development of fast and accurate diagnosis methods to ensure the best outcome for MD patients.

## Materials and Methods

### Ethical approval

All procedures followed in this study were in accordance with the ethical standards of the responsible committee on human experimentation (institutional and national) and with the Helsinki Declaration of 1964 and its later amendments. This project was approved by the Australian Genomics Health Alliance HREC/16/MH/251, and Sydney Children’s Hospitals Network Human Research Ethics Committee (HREC number MH2016.224). Written informed consent was obtained for this study. The study was approved by the Children’s Hospital of Eastern Ontario Research Ethics Board.

### Whole exome sequencing (WES)

Ion torrent sequencing of the mitochondrial genome did not reveal any pathogenic variants. Next-generation sequencing was performed at Randwick Genomics Laboratory (NATA accredited to ISO15189). Library preparation was performed using an Agilent SureSelect XT Low Input Clinical Research Exome x2 (Aligent CRE v2) kit, with libraries analysed on an Illumina NovaSeq 6000, at a multiplex of 90 exomes per S2 flow cell. Mean coverage was 146× with a minimum of 92% bases covered at least 20× yielding 120 000 variants from 8.4 × 10^7^ aligned reads. Data filtering was performed using an in-house pipeline (GAIA v. 3.2.5). The pipeline is based on Gemini V18 with annotation from VEP and dbNSFP and leverages family structures and known inheritance patterns.

Trio exome sequencing of the proband and parents from Family 3 was performed by next-generation sequencing in a clinical laboratory. Family 3 was enrolled into the Care4Rare research program in 2019 after the clinical trio exome did not identify any variants to explain the proband’s phenotype. The raw genomic data of these three family members were repatriated and processed through the Care4Rare Canada research bioinformatic pipeline to proceed to research reanalysis of the exome data (see Kernohan *et al.* (34) for technical details).

### Sanger sequencing

DNA from Subject 1 and control fibroblasts was extracted using a KAPA Express Extract Kit (Merck), and DNA was amplified using a KAPA2G Robust HotStart Ready Mix (Merck) according to the manufacturer’s instructions. Genomic DNA sequences around the *LYRM4* and *CRLS1* variant were amplified using the following primers:

**Table:** 

CRLS1-fwd	5′-CCC AGC CAT CTT CTG TAT TCT TG-3′
CRLS1-rev	5′-GAT TTG GAA GGA GAG CAA ACT GG-3′
LYRM4-fwd	5′-CCG AGC CGC AGC ATT TTA TTT C-3′
LYRM4-rev	5′-CCA CAC CTG TAG GCA GGA GCA C-3′

Sanger sequencing was performed by the Australian Genomics Research Facility (AGRF).

### Multiple sequence alignment and mitochondrial targeting sequence analysis

*CRLS1* and *LYRM4* amino acid sequences were sourced from UniProt (https://www.uniprot.org/) and were compared between species using ClustalW (https://www.ebi.ac.uk/Tools/msa/clustalw2/). Mitochondria targeting sequence cleavage site was determined using the Protein Subcellular Localization Prediction Tool (PSORT) (https://www.genscript.com/psort.html). Transmembrane domains were predicted using TMHMM 2.0 (https://services.healthtech.dtu.dk/service.php?TMHMM-2.0).

### Respiratory chain enzyme activity assays

Respiratory chain and citrate synthase enzymatic activities were measured in skeletal muscle and liver homogenates as previously described (20).

### Cell culture and transfections

Patient and control fibroblasts were grown in 37°C in humidified 95% air/5% CO_2_ in Dulbecco’s modified essential medium (DMEM) containing: glucose (4.5 g/l), l-glutamine (2 mm), fetal bovine serum (FBS) (10%, v/v), penicillin (100 U/ml), streptomycin sulphate (100 μg/ml) (Gibco, Life Technologies) and Normocin (Invitrogen), sodium pyruvate (1 mm) and uridine (50 μg/ml). Control fibroblasts were obtained from healthy paediatric individuals that were matched in age with the subjects. For transfections, cells were seeded at 80% confluency in the above media formulation without antibiotics and transfected with 263 ng/cm^2^ of wild-type *CRLS1* or *LYRM4* in a pCMV vector (Twist Bioscience) using FuGene HD (Promega) + Lipofectamine LTX (Invitrogen) (1:1) in OptiMEM (Invitrogen) and incubated for 48 h.

### Mitochondrial isolation

Mitochondria were isolated from cells by differential centrifugation using a buffer containing 250 mm sucrose, 1 mm EGTA and 5 mm Tris–HCl (pH 7.4) as previously described (35).

### Gel electrophoresis and immunoblotting

20 μg of isolated mitochondria from cultured cells were separated on a 4–16% Bis-Tris gel (Invitrogen). For Blue Native (BN)-PAGE, 80 μg of isolated mitochondria from cultured cells (solubilized in digitonin for supercomplex detection or *n*-dodecyl-β-d-maltoside (DDM) for individual complex detection) were separated on 3–12% Bis-Tris native gels (Invitrogen) as previously described (36). Samples from both denatured and native gels were transferred onto a PVDF membrane (Bio-Rad). Specific proteins were detected using mouse antibodies against, OXPHOS BN cocktail (ab110412), β-actin (ab6276) (Abcam, 1:1000), and rabbit antibodies against COXII (ab198286) (Abcam, 1:1000) in 20% Odyssey blocking buffer (Li-COR Biosciences) in Tris-buffered saline and 0.05% (v/v) Tween 20 (TBST). IRDye 680LT goat anti-mouse-IgG and IRDye 800CW goat anti-rabbit-IgG secondary antibodies (Li-COR Biosciences, diluted 1:10 000) were used to detected primary antibodies. Immunoblots were imaged using an Odyssey infrared imaging system (Li-COR Biosciences).

### Respiration

Mitochondrial oxygen consumption was measured in digitonin-permeabilized (20 μg/ml) cells, as previously described (37), using the Oxygraph 2k respirometer (Oroboros Instruments) in Mir05 buffer containing EGTA (0.5 mm), MgCl_2_.6H_2_0 (3 mm), lactobionic acid (60 mm), taurine (20 mm), KH_2_PO_4_ (10 mm), HEPES (20 mm), D-Sucrose (110 mm) and BSA, and essential fatty acid free (1 g/l), pH 7.1. Permeabilized cells were supplemented with 10 mm glutamate and 4 mm malate to measure ADP-independent respiration activity (state 4) of complex I followed by the addition of 1 mm ADP (Sigma) to measure ADP-dependent state 3 respiration, followed by the addition of 0.2 μm oligomycin. Complex IV-linked respiration was measured in permeabilized cells supplemented with 0.5 mm TMPD and 2 mm ascorbate in the presence of 5 μm antimycin to measure state 4 and 1 mm ADP (Sigma) was added to measure OXPHOS capacity or state 3 respiration, followed by 0.2 μm oligomycin. All data were normalized to mtDNA copy number, as done previously (14).

### DNA extraction and mitochondrial DNA copy number quantitative PCR

DNA was extracted from cells using a GeneJET Genomic DNA purification kit according to the manufacturer’s instructions (Fermentas, Thermo Fisher Scientific). Real-time PCR was conducted on 100 ng of DNA using previously described primers (5). Amplification was conducted using a Rotor-Gene-Q (Qiagen) using SensiMix SYBR mix (Bioline).

### Mitochondrial protein synthesis

Cells were seeded in six-well plates and allowed to attach overnight and de novo mitochondrial protein synthesis was measured using ^35^S radiolabelling as previously described (14). 17.5 μg of protein was separated on a 4–16% SDS-PAGE gel and equal loading was confirmed by Coomassie staining. Autoradiography signals were visualized on film using a Typhoon (GE Healthcare).

### Fluorescent microscopy

Cells were seeded on 22 mm^2^ glass coverslips and allowed to attach overnight. Cells were stained in 100 nm MitoTracker Orange (Molecular Probes, Thermo Fisher Scientific), fixed in 4% paraformaldehyde (w/v) in PBS, and mounted in DABCO/PVA medium as previously described (5). For rescue experiments, cells were transfected with 1 part GFP-tagged OXA1 in a pD2610-v10 vector to 4 parts experimental plasmid and imaging was conducted 48 h post-transfection. Images were acquired using a DeltaVision fluorescent microscope (GE Healthcare) using a 60× objective 1.58 NA oil immersion objective with a CoolSNAP HQ2 CCD Camera. All images are presented as maximum projections of 0.2 μm optical sections and deconvolution was performed by softWoRx software (GE Healthcare).

### Transmission electron microscopy

Cells were cultured in 35 mm Ibidi 500-grid plastic-bottomed μ-Dishes (Ibidi, Germany) for 48 h and then fixed in phosphate-buffered 4% PFA at 4°C overnight. The samples were post-fixed with 2.5% glutaraldehyde in 0.1 M sodium cacodylate buffer at 4°C overnight and then rinsed three times with 0.1 M sodium cacodylate buffer. All subsequent stages employed microwave-assisted processing in a BioWave Pro microwave system (Pelco) with previously reported settings (38). The samples were osmicated (1% (w/v) OsO_4_, 1.5% (w/v) K_3_Fe(CN)_6_ in 0.1 M cacodylate buffer (pH 7.4)), *en bloc* stained with 2% (w/v) aqueous uranyl acetate and then rinsed with distilled water before dehydration by graduated ethanol series (80%, 90%, 95%, 100%, 100% (v/v)) and propylene oxide (100%, 100% (v/v)). Sample infiltration with Araldite 502/Embed 812 was performed via graduated concentration series in propylene oxide under vacuum (25%, 50% 75% 100%, 100% (v/v)). The samples were polymerized at 60°C for 48 h and sectioned using a Leica UC6 ultramicrotome (Leica Biosystems) with a 45°C diamond knife (Diatome) to cut 75 nm ultrathin sections. The TEM grids were stained at room temperature using 2% (w/v) aqueous uranyl acetate (5 min) and Reynolds lead citrate (3 min) before routine imaging on a F200 FEGTEM (JEOL).

### Lipid extraction (Subject 1 p.(Ile109Asn) variant)

Samples (2 million cells each from control and patient fibroblasts) were lyophilized and then homogenized by adding 50–100 mg zirconium oxide beads (0.15 mm), 200 μl ice-cold 60% [v/v] methanol in water, 50 μl of a customized internal lipid standard mix in chloroform (including CL(14:0/14:0/14:0/14:0) and PG(15:0/18:1(d7))) and subsequently blending using a bullet blender (Next Advance) for 3 cycles of each 30 s at speed 8. Samples were frozen on dry ice in between cycles for 30 s. Then, to ensure complete homogenization, cells were incubated for 20 min in an ice-containing cold water using an ultrasonic bath. All solvents and mixtures contained 0.01% [w/v] butylated hydroxytoluene (BHT). Lipids were then extracted using a modified protocol published by Lydic *et al.* (39). In brief, 120 μl water (Milli-Q), 420 μl methanol and 220 μl chloroform were added to the homogenized cell pellet and mixed using the bullet blender. Samples were then centrifuged for 15 min at 20 000*g* and supernatants collected into 2 ml safe-lock Eppendorf tubes. The pellets were re-extracted using 100 μl water and 400 μl methanol–chloroform mixture (2:1 [v/v]) and re-homogenized in the bullet blender for 30 s at speed 8. Subsequently, samples were centrifuged for 15 min at 20 000*g* and then the supernatants pooled with the previous extracts. The pooled supernatants were dried using a speedVac evaporator and then reconstituted in 1 ml isopropanol:methanol:chloroform 4:2:1 [v/v/v] + 0.01% [w/v] BHT.

### Mass-spectrometry analysis for lipidomics (Subject 1 p.(Ile109Asn) variant)

100 μl of each lipid extract was dried in a 96 well plate (Eppendorf twin.tec PCR Plate 96, colourless) and then reconstituted in 50 μl isopropanol:methanol:chloroform 4:2:1 [v/v/v] containing 0.5 mm sodium acetate. The plate was sealed with Teflon Ultra-Thin sealing tape (Analytical Sales and Services, Pompton Plains, NJ). Samples were introduced to an Orbitrap Fusion Lumos ultra-high resolution/accurate mass spectrometer (Thermo Fisher Scientific) using an Advion Triversa Nanomate nano electrospray ionization (nESI) source (Advion). The nESI gas pressure was set to 0.3 psi and the spray voltage to 1.1 kV. The Orbitrap Fusion Lumos ion transfer capillary temperature was set to 150°C, the RF-value to 10%, the AGC target to 2 × 105 and mass resolving power at 500 000 (at 200 m/z). All spectra were recorded in negative ionization mode for a period of 3 min over a mass range of 350–1600 m/z. For cardiolipin (CL) analysis, a Field Asymmetric Ion Mobility Spectrometry (FAIMS) interface (FAIMS Pro, Thermo Fisher Scientific) was added to the Orbitrap Fusion Lumos and operated in standard resolution mode using a compensation voltage of 60 V (optimized for the transmission of the [M-2H]^2^- precursor ion charge states of cardiolipin species using authentic standards).

### Lipidomic data analysis (Subject 1 p.(Ile109Asn) variant)

Mass spectrometry–based lipidome data were analysed using a developmental version of Lipid Search 5.0 alpha software (Mitsui Knowledge Industry, MKI, and Thermo Fisher Scientific) with the following search parameters: Parent (mass) tolerance: ±3.0 ppm for PG and ±1.5 ppm for CL, Correlation threshold (%): 0.3, Isotope threshold (%): 0.1, Max isotope number: 1, Parent Threshold: 150, Recalc intensity threshold: 10 and Peak detection was set to profile and merge mode to average. The spectra were mass calibrated using internal standards. Lipid ‘sum composition’ assignments were achieved using an accurate mass-based, user-defined database. Automated peak finding and correction of ^13^C isotope abundances were performed by the Lipid Search 5.0 alpha software. Lipid nomenclature used is in accordance with the LIPID MAPS consortium proposed naming scheme (40,41). Semi-quantitative analysis of endogenous PG and CL species was achieved by comparing the peak areas of identified lipids of interest to the peak area of their respective internal standards (42). Lipidomics on fibroblasts and lymphocytes from Subject 2 was performed as previously described (22).

### Proteomics

Control and *Crls1^KO^* cell lines (*n* = 5 of each genotype) were prepared and proteomic analyses were carried out as described previously (30,43).

Patient and control fibroblasts (*n* = 5) were lysed in 5% SDS, 50 mm triethylammonium bicarbonate, 200 μg of protein was dissolved in 50 μl cell lysis buffer and digested using the S-trap mini-columns as per the manufacturer’s instructions. Briefly, dithiothreitol was added to a final concentration of 20 mm and samples were incubated at 70°C for 60 min. Proteins were alkylated by adding iodoacetamide to a final concentration of 40 mm and incubating at room temperature in the dark for 30 min. Proteins were acidified with 2.5 μl of 12% phosphoric acid and diluted with 150 μl of binding buffer (90% methanol, 100 mm final Tris). Samples were added to the S-Trap Mini Spin columns (Protifi) by centrifugation at 4000*g* for 30 s and then subsequently washed three times by successively loading 400 μl of binding buffer and centrifuging at 4000*g* for 30 s. Digestion was achieved by adding 1 μg sequencing-grade trypsin (Promega) and 125 μl of 50 mm triethylammonium bicarbonate and incubating for 1 h at 47°C. Peptides were eluted by successively adding 80 μl of 50 mm triethylammonium bicarbonate, 80 μl of 0.2% aqueous formic acid and 80 μl of 50% acetonitrile in 0.2% formic acid with a 30 s centrifugation step at 4000*g* between the addition of each elution buffer. The elutions were pooled, dried in a vacuum centrifuge and resuspended in 20 μl of buffer A (5% acetonitrile in 0.1% formic acid).

### Liquid chromatography and mass spectrometry for proteomics

Samples were analysed using a Thermo Fisher Scientific Ultimate 3000 RSLC UHPLC and an Eclipse mass spectrometer (Thermo Fisher Scientific). Samples were injected on a reverse-phase PepMap 100 C18 trap column (5 μm, 100 Å, 150 μm i.d. × 5 mm) at a flowrate of 10 μl/min. After 2.7 min, the trap column was switched in-line with a Waters nanoEase M/Z Peptide CSH C18 resolving column (1.7 μm, 130 Å, 300 μm i.d. × 100 mm) and the peptides were eluted at a flowrate of 0.9 μl/min buffer A (5% acetonitrile in 0.1% formic acid) and buffer B (80% acetonitrile in 0.1% formic acid) as the mobile phases. The gradient consisted of: 5–24% B for 0 to 22 min, 24–40% B from 22 to 35 min, 40–95% B from 35 to 39 min, followed by a wash, a return of 8% buffer B and equilibration prior to the next injection. The mass spectra were obtained in DIA mode with an MS1 resolution of 60 000, automatic gain control target at 200%, maximum injection time at 40 ms and scan range from 350 to 1200 m/z. DIA spectra were recorded at resolution 15 000 and an automatic gain control target of 800%. The 70 isolation windows were 10 m/z each from mass 399.9319 to 1101.2502.

### DIA mass spectrometry data analysis

Data analysis was performed with Spectronaut (44) using direct DIA analysis and default settings. Briefly, spectra were searched against the *Homo sapiens* proteome database from UniProt with carbamidomethylation set as a fixed modification and methionine oxidation and N-terminal acetylation as variable with 1% false discovery rate (FDR) cutoffs at the peptide spectral match, peptide and protein group levels. Quantitation was performed at the MS2 level with *Q*-value data filtering and cross-run normalization with Q-complete row selection. GO term analysis was performed using DAVID v6.8 (45,46), with significant enrichment of GO terms determined at FDR < 0.1. Volcano plots for mitochondrial proteins were generated using OmicsVolcano (47).

### Statistical analysis

Unless otherwise specified, data are presented as mean ± standard error of the mean (SEM), and statistical significance was determined using a Student’s *t* test.

## Supplementary Material

Lee_et_al_Supplementary_Information_revised_ddac040

Supplementary_Data_ddac040

## Data Availability

The CRLS1 proteomic datasets were submitted to PRIDE (PXD029861). The WES datasets from this work have not been deposited in a public repository because of ethical restriction but are available from the corresponding author and colleagues on request.
