# Correction to: Deleterious variants in *CRLS1* lead to cardiolipin deficiency and cause an autosomal recessive multi-system mitochondrial disease

**DOI:** 10.1093/hmg/ddac294

**Published:** 2022-12-22

This is a correction to: Richard G Lee, Shanti Balasubramaniam, Maike Stentenbach, Tom Kralj, Tim McCubbin, Benjamin Padman, Janine Smith, Lisa G Riley, Archana Priyadarshi, Liuyu Peng, Madison R Nuske, Richard Webster, Ken Peacock, Philip Roberts, Zornitza Stark, Gabrielle Lemire, Yoko A Ito, Care4Rare Canada Consortium, Kym M Boycott, Michael T Geraghty, Jan Bert van Klinken, Sacha Ferdinandusse, Ying Zhu, Rebecca Walsh, Esteban Marcellin, David R Thorburn, Tony Roscioli, Janice Fletcher, Oliver Rackham, Frédéric M Vaz, Gavin E Reid, Aleksandra Filipovska, Deleterious variants in *CRLS1* lead to cardiolipin deficiency and cause an autosomal recessive multi-system mitochondrial disease, Human Molecular Genetics, Volume 31, Issue 21, 1 November 2022, Pages 3597–3612, https://doi.org/10.1093/hmg/ddac040

In the originally published version of this manuscript, the names of authors Tony Roscioli and Ying Zhu were inadvertently misspelled.

These errors have been corrected online.

