# Supplementary material for: Deleterious variants in CRLS1 lead to cardiolipin deficiency and cause an autosomal recessive multi-system mitochondrial disease
Source: Hum Mol Genet. 2022 Feb 11;31(21):3597–612. doi: 10.1093/hmg/ddac040 (PMC9616573; doi:10.1093/hmg/ddac040)
Supplement: Lee_et_al_Supplementary_Information_revised_ddac040 [file lee_et_al_supplementary_information_revised_ddac040.zip › Lee_et_al_Supplementary_Information_revised_ddac040.docx]

**Supplementary Figures**

**
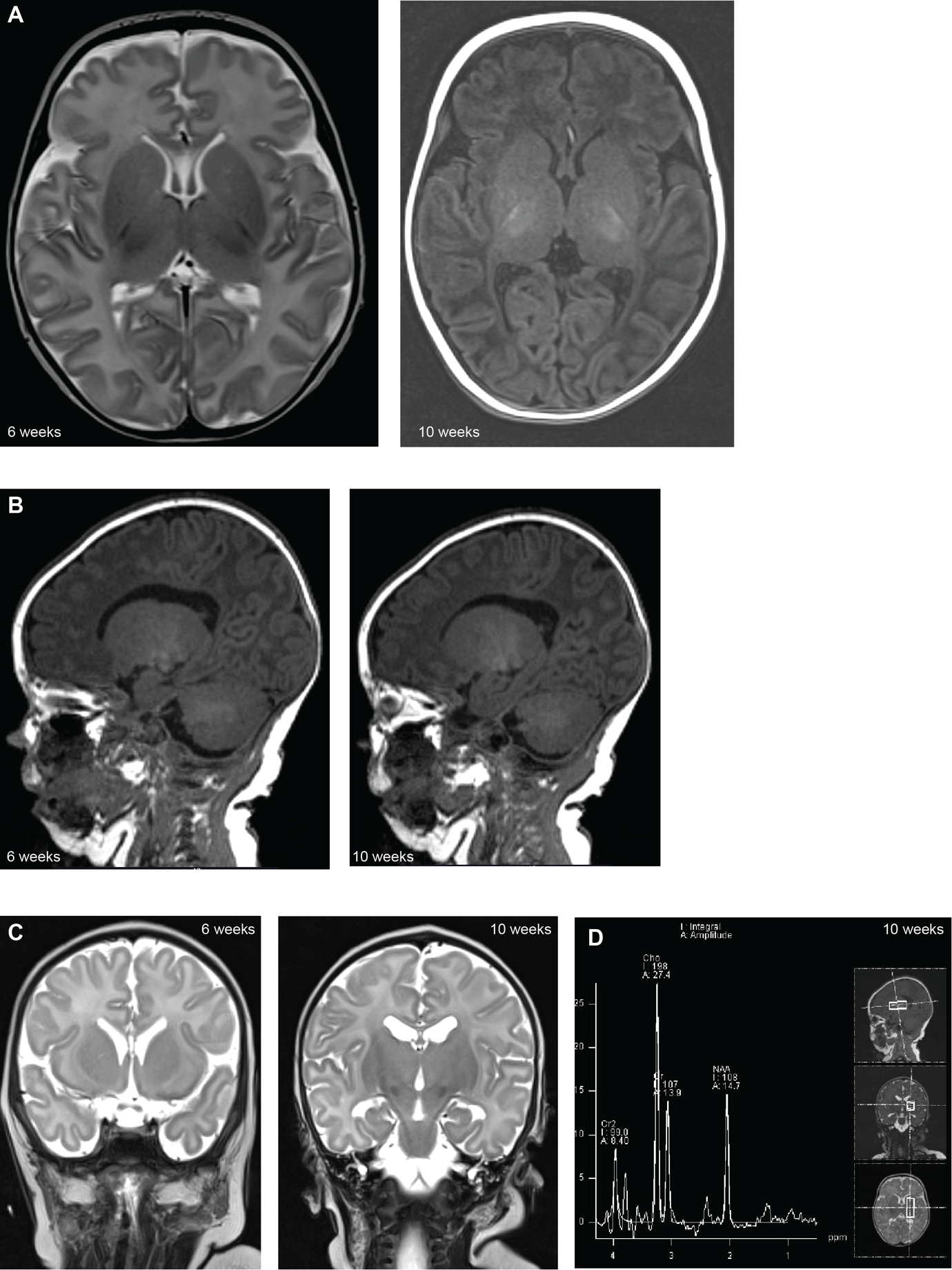
**

**Supplementary Figure 1.** Brain magnetic resonance imaging (MRI), (**A**) axial, (**B**) sagittal and (**C**) coronal views of Subject 1, respectively, at 6 and 10 weeks. (**D**) Brain magnetic resonance spectroscopy (MRS) of Subject 1 at 10 weeks.

**
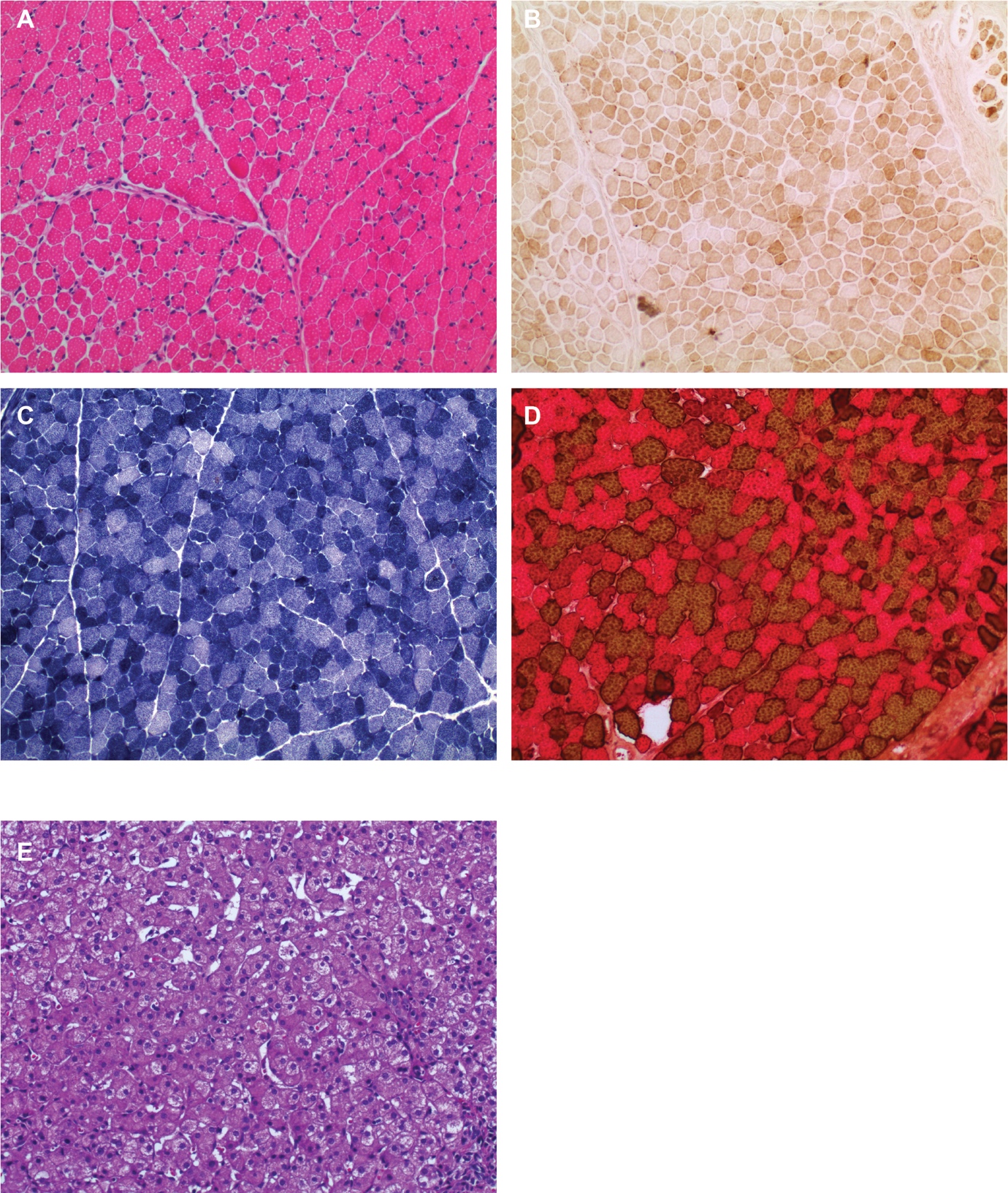
**

**Supplementary Figure 2.** Skeletal muscle histology of Subject 1. (**A**) Hematoxylin and eosin (H&E) stain showing variation in fibre size with the presence of scattered small fibres. (**B**) Cytochrome c oxidase (COX) and succinate dehydrogenase (SDH) double staining showed no evidence of COX deficient fibres. (**C**) NADH staining was normal. (**D**) Muscle immunoperoxidase stain showed normal muscle fibre typing, with a slight preponderance of type 1 (slow-twitch, stains red) presence compared to type 2 (fast twitch, stains brown) fibres. All images have magnification of 20x. (**E**) Liver H&E staining of hepatocytes showed some granularity and possible ballooning with microvesicular steatosis. Magnification is 20x.

**
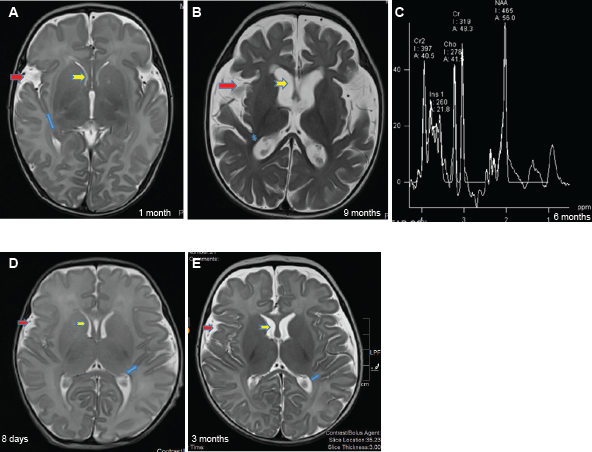
**

**Supplementary Figure 3.** Brain MRI, axial T2 views of Patient II-1, Family 2, at one month (**A**) and nine months of age (**B**). There is evidence of progressive global cerebral atrophy with an increase in ventricular volumes (yellow arrows), an increase in extra-axial CSF spaces (red arrows) and a decrease in the white matter volume (double headed blue arrows). (**C**) Brain MRS at 6 months was normal. Brain MRI, axial views of Patient II-3, Family 2, at day 8 was normal (**D**), but repeat study at 3 months (**E**) showed subtle loss of brain volume with an increase in ventricular size (yellow arrows), an increase in the volume of extra-axial CSF spaces (red arrows) and white matter volume loss (double headed blue arrows).


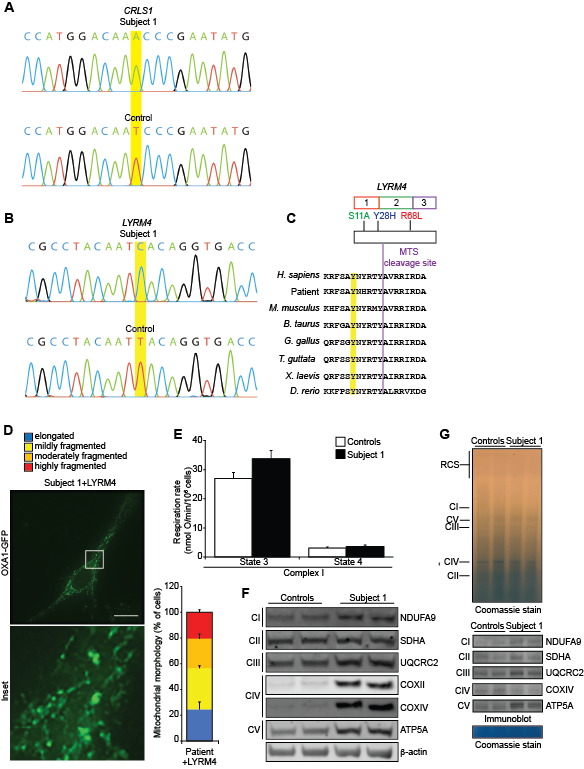


**Supplementary Figure 4.** (**A**) Sanger sequencing identifying novel variants in *CRLS1* and *LYRM4* (**B**) in Subject 1. (**C**) Schematic representation of the *LYRM4* gene and its encoded protein showing the mitochondrial targeting sequence cleavage site. The variant amino acid is coloured blue, with other *LYRM4* variants previously shown to be pathogenic (red) and benign variants (green) also annotated. Multiple sequence alignment for LYRM4, where the variant amino acid location is highlighted in yellow. (**D**) Mitochondrial morphology of Subject 1 cells expressing wild-type LYRM4 and visualised by examining co-expressed OXA1-GFP fluorescence, showed that transient LYRM4 expression did not alter Subject 1 cell morphology. Qualitative scoring of mitochondrial morphology (n>50) is shown for all cells. (**E**) Oxygen consumption of Complex I under phosphorylating (state 3) and non-phosphorylating (state 4) conditions was measured in three controls and Subject 1 fibroblasts using an OROBOROS high-resolution respirometer and the results are representative of three biologically independent experiments. (**F**) SDS-PAGE and immunoblotting on 30 µg of isolated mitochondria from two controls and Subject 1 fibroblasts. (**G**) BN-PAGE of 80 µg of isolated mitochondria from two controls and Subject 1 fibroblasts and immunoblotted to detect OXPHOS complexes I-V. All data are representative of at least three biologically independent experiments.

**
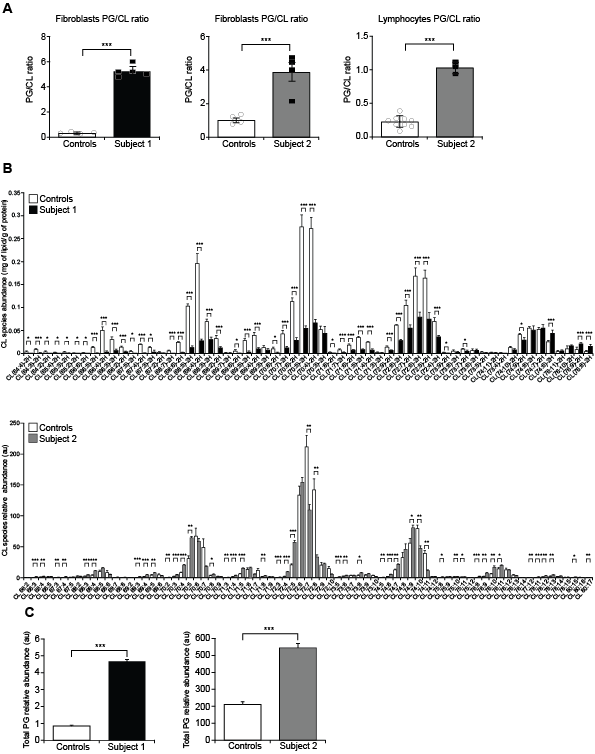
Supplementary Figure 5.** (**A**) Ratio of PG:CL in controls, Subject 1 and Subject 2 fibroblasts and Subject 2 lymphocytes. Data are ratios shown as mean ± SEM. FDR<0.001, ***. (**B**) Cardiolipin acyl chain profiles of Subject 1 and 2 relative to control fibroblasts (n=4). Data are absolute values shown as mean ± SEM. FDR<0.05, *; FDR<0.01, **; FDR<0.001, ***. (**C**) PG levels in controls, Subject 1 and 2 fibroblasts (n=4). Data are absolute values shown as mean ± SEM. FDR<0.001, ***.
